# Age-related variations in physical activity, happiness, and psychological well-being: Evidence from Iran

**DOI:** 10.1371/journal.pone.0314202

**Published:** 2025-01-06

**Authors:** Mohammad VaezMousavi, Lara Carneiro, Amir Shams, Hamed Abbasi, Parvaneh Shamsipour Dehkordi, Mahdi Bayati, Hadi Nobari

**Affiliations:** 1 Imam Hossein University, Tehran, Iran; 2 Physical Education Department, College of Education, United Arab Emirates University, Al Ain, Abu Dhabi, United Arab Emirates; 3 Department of Motor Behavior, Sport Sciences Research Institute, Tehran, Iran; 4 Department of Sports Medicine, Sport Sciences Research Institute, Tehran, Iran; 5 Department of Motor Behavior, Faculty of Sport Sciences, Alzahra University, Tehran, Iran; 6 Department of Exercise Physiology, Sport Sciences Research Institute, Tehran, Iran; 7 Faculty of Sport Sciences, University of Extremadura, Caceres, Spain; Alexandria University Faculty of Nursing, EGYPT

## Abstract

**Background:**

Physical activity (PA) is associated with higher levels of PWB and happiness. The high prevalence of physical inactivity among the Iranian population is concerning. Moreover, according to the Happiness Report 2024, Iran is ranked 100th out of 143 countries. Thus, this research aimed to study the PWB, happiness, and PA in Iranian age groups.

**Methods:**

The study sampled 1,050 participants, equally divided into young, middle-aged, and elderly groups, using randomized cluster sampling from urban areas across five geographic regions in Iran. The study utilized four questionnaires: a Demographic Information Questionnaire, the International Physical Activity Questionnaire (IPAQ), Ryff’s Scales of PWB (18-item form), and the Oxford Happiness Questionnaire (OHQ). Descriptive statistics were used for summarizing data, and inferential analyses included Pearson’s correlation coefficient, one-way ANOVA, the Bonferroni test, and multivariate regression, all with a significance level set at P<0.05.

**Results:**

Middle-aged individuals had the highest PA (1015.69±730.63 MET-minutes per week) and reported the highest happiness (111.97±24.08) and PWB (63.75±11.17) levels. The elderly had the lowest levels of PA (677.78±592.50 MET-minutes per week), happiness (107.63±24.01), and PWB (60.05±10.02). Statistical tests showed significant differences in PA, happiness, and PWB between the age groups (P<0.05), with no significant difference between young and elderly in PA (P>0.05). Physical activity positively correlated with happiness and PWB, especially in the elderly. Multivariate regression revealed that PA significantly predicts happiness (F = 30.993, t = 6.96, B = 0.211) and PWB (F = 86.85, t = 9.32, B = 0.004), explaining 4.4% and 8.0% of their variances, respectively.

**Conclusions:**

The study concludes that increasing PA enhances happiness and PWB. Practical applications include promoting PA to improve mental health and PWB. Future research should investigate the effects of specific types of PA and explore longitudinal impacts on PWB to provide a deeper understanding of these relationships.

## 1. Introduction

Psychological well-being (PWB) is a central theme in the humanistic psychology literature [1], encompassing various aspects of welfare. These include positive self-assessment and evaluation of one’s past life, known as self-acceptance, and a sense of ongoing personal growth and development, referred to as personal growth. It also involves the belief in leading a purposeful and meaningful life (purpose in life), the presence of warm, fulfilling, and trusting relationships with others (positive relations with others), the ability to effectively manage one’s life and environment (environmental mastery), and a sense of self-determination [2].

Happiness is often seen as a critical indicator of societal development and overall well-being worldwide and is recognized as a measure of social progress. It is often used as a critical objective in policy-making across various countries [3]. The World Happiness Report 2024 shows that Finland, with a score of 7.741, is the happiest country in the world, while the Islamic Republic of Iran is ranked 100th with a score of 4.923. Afghanistan is ranked 143rd with a score of 1.721, making it the unhappiest country according to the report [4]. Average happiness levels vary across countries and communities, likely due to differences in social characteristics [5] and living standards [6]. Cultural differences also play a significant role in determining these variations in happiness [7].

A cross-sectional survey, which aimed to assess the level of happiness in Iran’s work communities, was conducted with 13,842 randomly selected employees from 380 workplaces across 31 provinces. Data was collected through structured questionnaires, and the analysis revealed that the average happiness score across Iran’s work communities was 141.22±22.89. The highest happiness level was observed in Boushehr Province (148.97±21.49), while the lowest was in Hormozgan Province (130.39±25.28). The findings suggest that enhancing job security, fostering positive interactions, improving management, increasing salaries and benefits, and offering more flexible working hours are all strategies that can increase happiness. Empowering employees, involving them in decision-making, and sharing organizational profits can further enhance worker happiness and loyalty [8].

Another recent study explored the determinants of happiness in Shiraz, Iran. The average happiness score among participants was 6.41 out of 10, with lower levels observed in older adults, illiterates, the unemployed, and those separated from their spouses [3].

Another study measured the level of happiness in Kerman, Iran, using the Oxford Happiness Inventory (OHI) and a self-report questionnaire. The findings showed that around 90% of participants reported moderate to high happiness levels, but the mean happiness score was relatively low at 43.2. Factors such as illiteracy, unemployment, divorce, living in deprived areas, stress, weak religious beliefs, lower income, and poor health significantly decreased happiness levels. The study suggests that interventions focused on improving community health, stress management, and access to urban facilities could enhance happiness in the population [9].

Studies have shown that PA is associated with increases in many aspects of well-being, such as happiness, life satisfaction, and mood regulation [10–12]. For example, one study explored the impact of regular PA on happiness, life satisfaction, and self-esteem over four weeks. Participants were divided into three groups: active, beginners, and inactive. Results showed that active individuals reported higher levels of happiness, self-esteem, and life satisfaction than beginners and inactive participants. Additionally, beginners experienced increased life satisfaction and happiness after four weeks of exercise. The findings confirm that regular PA significantly enhances subjective well-being, even with a short duration of engagement [13].

According to the World Health Organization’s (WHO) STEPwise approach to risk factor Surveillance (STEPS) 2021, the prevalence of insufficient physical activity (IPA) was significantly high in Iran [14]. This study evidenced that the prevalence of IPA in Iranian adults above eighteen years of age had roughly doubled from 2001 to 2016 and that more than half the population (about 55%) had IPA in 2016. Also, 5% of mortalities in 2017 in Iran were caused by IPA.

One recent cross-sectional study reported that 55.1% of Iranians showed low or moderate levels of happiness, and those who engaged in active leisure pursuits reported significantly higher levels of happiness than those who were inactive during leisure time. The authors suggest that engaging in PA during leisure time is a predictor of happiness [15]. Another cross-sectional study was conducted on a sample of adults aged 18–65 in Iran in 2020 to assess the association between happiness and self-rated health. The results showed that self-rated health was the most significant factor that affected happiness, even after adjustment for socioeconomic variables, income, employment, age, and education. Improving population health might be an effective measure to improve happiness among Iranians [16].

This study aims to provide a clearer perception of the current state of the nation’s mental health by examining the relationship between PA and psychological variables associated with a healthy life. The findings from this study can guide policymakers in designing interventions aimed at enhancing the health and happiness of individuals living in Iran.

## 2. Methods

### 2.1. Participants

The study involved 1,050 individuals divided into three age groups: young, middle-aged, and elderly, with 350 participants in each group. These individuals were selected through randomized cluster sampling from five geographic regions of the country, namely East, West, North, South and the Center. It is important to note that the research focused exclusively on urban areas, as the pursuit of PA and sports in cities has become a significant concern for public health and social policy.

Each geographic region was treated as a cluster representing a province, and participants were randomly chosen from the centers of these selected provinces. To be eligible, participants had to demonstrate a willingness to provide informed consent and participate in assessments. They were required to self-report their general health status as either “good” or “fair”. Individuals with diagnosed psychiatric disorders, such as severe depression or bipolar depression, as well as those with chronic illnesses or disabilities that significantly limit PA (including cardiovascular diseases) were excluded. Additionally, participants who had been hospitalized or undergone major surgery in the six months before the study, or who had a history of substance abuse disorders that impacted PA and PWB were excluded.

### 2.2. Ethics

This research was conducted under the supervision of the Ethics Committee at the Sport Sciences Research Institute of Iran, with the approval code IR.SSRI.REC. 952694. Before commencement, all participants were informed about the study’s objectives and methodology. Participation was entirely voluntary, and all subjects signed an informed consent form before participating. Participants’ privacy rights were strictly upheld throughout the process. The research adhered to ethical principles outlined in the Helsinki Declaration. The recruitment period for the study spanned from June 20, 2016, to October 10, 2016.

### 2.3. Measures

The following questionnaires were used to collect information:

#### 2.3.1. Demographic information questionnaire

This questionnaire collected demographic data, including age, gender, education level, marital status, place of residence (central, northern, southern, western, and eastern regions of Iran), and type of sport.

#### 2.3.2. International Physical Activity Questionnaire (IPAQ)

The IPAQ assessed PA levels among individuals aged 15 to 69. It has a multidisciplinary framework that evaluates PA in various domains, such as work, travel, leisure, household chores, and yard work, categorizing activities into low, moderate, and severe intensity. The structured protocol and consistent scoring of the IPAQ facilitate robust activity comparisons across different studies. The questionnaire consists of 7 sections and 27 questions, with participants reflecting on their activity levels over the past seven days before answering [17]. The IPAQ’s reliability coefficients in this study were 0.66 and 0.61, determined using Cronbach’s alpha and split-half methods, respectively.

#### 2.3.3. Ryff’s scales of psychological well-being

Initially developed by Ryff in 1980, this scale comprised 120 questions. However, subsequent research has produced shorter versions with 84, 54, and 18 items. This study’s 18-item version was employed, which includes six sub-scales: self-acceptance, positive relationships with others, autonomy, purposeful life, personal growth, and environmental mastery. Each sub-scale question uses a 6-point Likert scale, yielding a score of 1 to 6, where higher scores indicate improved PWB [2]. The scale’s reliability in the current study was reported as 0.73 and 0.65, as measured by Cronbach’s alpha and split-half methods, respectively.

#### 2.3.4. Oxford Happiness Questionnaire (OHQ)

This questionnaire was initially presented by Argyle et al. [18] as the Oxford Happiness Inventory (OHI), which had 29 questions and was based on the Beck questionnaire. Hills and Argyle [19] reviewed the inventory, modified the rates for each question, including the reverse questions, and changed its name to the Oxford Happiness Questionnaire. In this study, the questionnaire scoring changed from four to six points. The authors found that the validity and reliability of this questionnaire were 0.56 and 0.95, respectively. In the present study, the reliability of this scale was 0.89 and 0.84, respectively, using Cronbach’s alpha and split-half methods.

### 2.4. Data collection method

To conduct the study, two districts were selected from each provincial center. Within these districts, two parks were designated as sites for administering and collecting questionnaires. Two trained interviewers were appointed for each city, each possessing specialized skills in engaging with diverse age groups and administering questionnaires. For participants who required assistance in completing the questionnaires, particularly elderly individuals, the interviewers read the questions aloud and provided aid in formulating their responses. The data collection phase occurred from June 20, 2016 to October 10, 2016.

### 2.5. Statistical analysis

Descriptive statistics were utilized to summarize the data, providing a clear overview of the demographic characteristics and responses of the participants. Inferential analyses were performed to explore relationships and differences among the variables. Pearson´s correlation coefficient was used to assess the strength and direction of relationships between continuous variables, which are essential to understand how different aspects of PA and PWB are interconnected.

One-way ANOVA was employed to compare means across the three age groups, allowing for the identification of significant differences in PA levels and PWB among the young, middle-aged and elderly participants. The Bonferroni test was then applied as a post-hoc for multiple comparisons between groups. Multivariate regression analysis was conducted to examine the impact of various predictors on outcomes related to PA and PWB. All statistical analyses were performed using SPSS, with a significant level set as P<0.05.

## 3. Results

The age-related findings revealed that the average ages for young, middle-aged, and elderly individuals were 20.8±4.1, 46.7±6.1, and 70.1±5.1, respectively. Data on PA, happiness, and PWB for all groups can be found in Table 1.

**Table 1:** Mean, standard deviation, and 95% confidence interval of measured variables in the studied groups.

Age groups	Physical activity (MET-minutes per week)	Happiness	Psychological well-being
Young	710.99±569.85 [651.08–770.89]	104.94±22.80 [102.54–107.34]	58.82±8.91 [57.88–59.75]
Middle-aged	1015.69±730.64 [938.88–1092.50]	111.97±24.08 [109.44–114.50]	63.75±11.17 [62.57–64.92]
Elderly	677.79±592.50 [615.50–740.07]	107.63±24.65 [105.04–110.22]	60.05±10.02 [58.99–61.10]

Values are given as mean ± SD [95% confidence interval]. MET, Metabolic Equivalent

The middle-aged group exhibited the highest level of PA, while the elderly had the lowest. Additionally, the results indicate that the middle-aged individuals reported the highest levels of happiness and PWB, whereas young individuals showed the lowest levels, as shown in Table 1.

To assess normality, the Shapiro-Wilk test was conducted for each group. Homogeneity of variances was evaluated using Levene´s test. These assumptions were verified to ensure the appropriate application of the one-way ANOVA and subsequent analyses.

From the analysis presented in Table 2, the results of one-way ANOVA demonstrated a significant difference in mean activity levels among the groups (*F*(2,1047) = 30.111, *P* = 0.001). To further examine group differences, Bonferroni’s test was conducted. The outcomes revealed that the PA level of middle-aged individuals was significantly different from that of both elderly and young individuals (P<0.05), indicating that middle-aged individuals had a higher activity level compared to the other groups. However, no significant difference in PA was found between young and elderly people (P>0.05).

**Table 2:** Comparison of physical activity, happiness, and psychological well-being among age groups.

Variables		Sum of Square	df	Mean of square	F	P
Physical activity	Between groups	24281609.80	2	12140804.900	30.111	0.001*
	Within groups	422156870.46	1047	403206.180		
	Total	446438480.26	1049	--------		
Happiness	Between groups	8810.710	2	4405.355	7.742	0.001*
	Within groups	595742.90	1047	569.001		
	Total	604553.60	1049	--------		
Psychological well-being	Between groups	4612.95	2	2306.472	22.721	0.001*
	Within groups	106285.40	1047	101.514		
	Total	110898.35	1049	--------		

*Significant level at P<0.05

The results of the one-way ANOVA test in Table 2 also indicated that there is a significant difference between the averages of happiness (*F*(2,1047) = 7.742, *P* = 0.001) and PWB (*F*(2,1047) = 22.721, *P* = 0.001) in the studied groups. Thus, Bonferroni’s test was used to assess the group differences. Results showed that there is a significant difference between the happiness and PWB levels of the middle-aged compared with the elderly and young people (P<0.05). Middle-aged people had a higher level of happiness and PWB than the elderly and young. However, the difference between the happiness and PWB of the young and the elderly is not significant (P>0.05).

Physical activity scores do not correlate with the happiness of young people (r = 0.063, *P* = 0.241), but there is a poor correlation (r = 0.136, *P* = 0.011) with the happiness of middle-aged people and a stronger correlation with the happiness of the elderly (r = 0.247, *P* = 0.001) In other words, increasing PA increases the happiness of all age groups (P<0.05). Results also showed that there is a positive and significant correlation between the mean scores of PA and PWB in the age groups and increasing the level of PA increases the rate of PWB in all age groups (P<0.05).

Multivariable regression was used to determine the rate of predicting the criteria variables (happiness and PWB) based on the predictor variable of PA in the age groups. As shown in Table 3, the observed F value is significant to estimate happiness according to the PA levels (P = 0.001). The effective coefficient of the PA variable (t = 6.9602 and B = 0.21095) also shows that PA with 99.9% confidence can predict changes in happiness. However, the result of the coefficient of determination shows that only 0.044% of the variance in happiness is explained by PA.

**Table 3:** The regression model of physical activity effect on happiness and psychological well-being.

**Variable**	**Model**	**SS**	**df**	**Ms**	**F**	**P**	**R**	**R** ^**2**^	**SE**
Happiness	Regression	17365.21	1	17365.21	30.993	0.001	0.169	0.029	23.67
	Residual	587688.4	1048	560.29					
Psychological well-being	Regression	8487.76	1	8487.76	86.85	0.001	0.28	0.08	9.88
	Residual	102410.6	1048	97.72					
**Variable**		**B**		**SEB**	**Beta**	**T**		**P**	
Happiness	Constant	103.182		1.158	--------	89.142		0.001	
	Physical activity	0.006		0.001	0.169	5.567		0.001	
Psychological well-being	Constant	57.37		0.48	--------	118.68		0.001	
	Physical activity	0.004		0.001	0.28	9.32		0.001	

Finally, the results of the regression test showed that the observed F value is significant in predicting the rate of PWB based on PA (P = 0.001). The effective coefficient of the PA variable (t = 9.32 and B = 0.004) also shows that PA with 99.9% confidence can predict the changes related to PWB. The results of the coefficient of determination indicate that only 0.08% of the variance in PWB is explained by PA.

## 4. Discussion

Humanity has long pursued the goal of longevity, and many countries now anticipate that people will live over 80 years. The World Population Prospects 2019 [20], projects that by 2050, 1 in 6 people in the world will be over the age of 65, up from 1 in 11 in 2019 [20]. Iran has experienced a significant decrease in mortality rates over the last 40 years and boasts one of the highest life expectancies in the Middle East, with females reaching 81.6 years and males 76.1 years in 2019 [21]. However, this longevity does not necessarily translate into better health [22]. Physical activity is recommended as a key lifestyle behavior for managing various chronic conditions globally [23] and has been identified as a key factor influencing people’s well-being [10, 13]. However, one study that examined the prevalence and patterns of IPA across Iran in 2021, found a high overall IPA prevalence of 51.3%, with higher rates among women (57.87%) compared to men (41.93%). The study identified significant regional variations, with Yazd province having the highest IPA rate (63.45%) and West Azerbaijan the lowest (39.53%). Factors such as living in urban areas, marital status, and higher wealth index were associated with higher IPA rates. The findings highlight the need for targeted interventions to increase PA, particularly in urban settings and among women. Our results demonstrated significant differences in PA levels between middle-aged individuals and both the elderly and younger people, with middle-aged individuals being more active. It also showed that the elderly had the lowest level of PA.

These results are in line with the findings from STEPS 2021 which showed that the prevalence of IPA is high in Iran, especially among females and the elderly population [24].

In this context, various obstacles to PA among the elderly in Iran and globally can be grouped into three main categories: interpersonal (such as the absence of a companion, lack of professional guidance, family obligations, and social pressures), intrapersonal (including physical health issues, time constraints, lack of interest, laziness, financial costs, security concerns, and fear of falling), and environmental factors (like traffic, weather conditions, and physical barriers to walking) [25]. Moreover, the results of epidemiological studies [26] corroborated that PA decreases from teens to the elderly, and this decline is one of the public health problems in most countries of the world [27, 28]. Physical inactivity is common among older adults and can negatively affect chronic medical conditions, cognitive function, and physical abilities, leading to reduced quality of life and increased mortality [29]. Therefore, it is crucial to implement effective interventions encouraging PA among the elderly to improve their quality of life and prevent diseases.

The results also showed significant differences in happiness and PWB across different age groups. Middle-aged individuals reported higher levels of both happiness and PWB compared to the elderly and young people (P<0.05). In contrast, no significant difference was observed between the young and elderly (P>0.05).

This is not in agreement with the results from the World Happiness Report 2024 [4], which compared four groups in Iran (young, lower middle, upper middle, and elderly). The report found that the young were the happiest, while the elderly were the least happy. Additionally, in the twenty countries of the Middle East and North Africa, happiness is highest among the young, particularly young females, and then decreases gradually before increasing again in females aged 60 and over. The region shows considerable diversity; in Israel, the younger population experiences the highest levels of happiness, while in the United Arab Emirates and Saudi Arabia, where there are many foreign-born workers in the younger age groups, the pattern is reversed.

A recent study underscored the rapid growth of the elderly population in Iran and the significance of social support in enhancing their happiness [30]. Another study stated that in Iran the prevalence of older people living alone is growing due to changes in family structure and the effects of culture and tradition [31]. Additionally, this rise in life expectancy, which increases the number of years of life with a disease or disability, will generate more difficult conditions for older people living alone. Furthermore, Iran is positioned at **100th** overall out of 149 countries in the 2024 World Happiness Report, but the report specifically highlights that younger individuals (aged below 30) in Iran are ranked at **96th**, while older adults (aged 60 and above) are placed at **103**^**rd**^ [4].

Results also showed that PA correlated significantly with happiness and PWB in young, middle-aged and elderly people, with a stronger correlation in the last group. Consistent with these results, a recent cross-sectional study developed in Iran revealed that individuals who participated in active leisure pursuits experienced significantly higher happiness levels than those who were inactive during their leisure time. The authors suggest that engaging in PA during leisure time predicts happiness [15].

In accordance, available evidence confirms a positive relationship between PA and happiness [32, 33]. For example, one study systematically reviewed the literature to explore the relationship between PA and happiness. After searching major databases for relevant studies published post-1980, 23 studies were analysed, including 15 observational and 8 intervention studies. All observational studies reported a positive association between PA and happiness, with even minimal activity (e.g., 10 minutes per week) linked to increased happiness. Two studies suggested that this relationship might be mediated by health and social functioning. While randomized controlled trials, mostly involving older adults and cancer survivors, showed that exercise improved happiness, the limited number of such trials prevents firm conclusions about causality [34]. Interestingly, the strongest correlation between happiness, PWB and PA was seen in the elderly group.

One study developed in Iran demonstrated that aerobic exercise improved PWB and quality of life in older adults, with moderate-intensity exercise seeming to produce higher benefits than low-intensity, establishing a positive dose-response relationship [35].

The multivariate regression analysis revealed that PA is a statistically significant predictor of both happiness (P = 0.001, t = 6.96, B = 0.211) and PWB (P = 0.001, t = 9.32, B = 0.004). However, PA only accounts for 0.044% of the variance in happiness and 0.08% of the variance in PWB. Thus, while higher levels of PA are statistically linked to happiness and PWB, the practical significance of these findings is minimal.

Notably, in the last thirty years, systematic reviews [36], meta-analyses [37], and umbrella reviews [38] have contributed to the understanding of the impact of PA on mental health and well-being [39]. Therefore, PA and exercise have been recognized as effective components of treatment for various mental disorders [36, 40–42].

Our results suggest that happiness cannot be separated from a broader construct of PWB and physical and mental health, and confirm an association between PA and happiness [43]. Understanding the relationships between happiness, PA, and PWB and mental health symptoms is crucial for informing social policy decisions.

It is important to note that this study has some limitations. It focused solely on urban areas, excluding rural regions. This limits the findings’ generalizability to rural populations with different PA patterns, happiness and PWB levels. The study groups (young, middle-aged, elderly) may not fully account for the nuances within each age category. For instance, variations within the "middle-aged" or "elderly" categories could influence the results. The exclusion of participants with mental disorders might not capture the full range of PWB and happiness levels, especially as these disorders are prevalent in all age groups. The reliance on self-reported questionnaires could introduce biases or inaccuracies in reporting PA levels, happiness, and PWB. Finally, the results are limited in their generalizability as the data was collected exclusively in Iran. This geographic specificity may not reflect the PA patterns, happiness levels, or PWB of individuals in other countries or regions with different cultural, social, or economic contexts.

The findings underscore the need for targeted urban health interventions and policies in Iran, particularly those aimed at enhancing PA and PWB across different age groups. Longitudinal studies within Iran could provide valuable insights into how PA and PWB evolve over time and in response to various interventions.

## 5. Conclusions

The results indicate that middle-aged individuals exhibit higher levels of PA, PWB, and happiness than the elderly and younger populations. Furthermore, happiness, PWB, and life satisfaction were positively correlated with PA scores across all groups. Our findings suggest that higher levels of PA are statistically linked to happiness and PWB. This research provides valuable insights for future public health initiatives to enhance happiness and foster better physical and mental health. Prioritizing PA in public health strategies could improve happiness and PWB for all age groups, with particular benefits for the elderly.

## Supporting information

S1 Data (XLSX)
